# Correlates of never testing for HIV among men who have sex with men in Malaysia: A cross-sectional study

**DOI:** 10.1371/journal.pone.0294937

**Published:** 2023-11-30

**Authors:** Luzan JadKarim, Jeffrey Wickersham, Kamal Gautam, Iskandar Azwa, Rumana Saifi, Antoine Khati, Kiran Paudel, Toan Ha, Roman Shrestha

**Affiliations:** 1 Yale School of Medicine, Department of Internal Medicine, Section of Infectious Diseases, New Haven, CT, United States of America; 2 Department of Allied Health Sciences, University of Connecticut, Storrs, CT, United States of America; 3 Department of Medicine, Faculty of Medicine, Centre of Excellence for Research in AIDS, University of Malaya, Kuala Lumpur, Malaysia; 4 Nepal Health Frontiers, Kathmandu, Nepal; 5 School of Public Health, University of Pittsburgh, Pittsburgh, PA, United States of America; HIV/STI Surveillance Research Center and WHO Collaborating Center for HIV Surveillance, Institute for Future Studies in Health, Kerman University of Medical Sciences, ISLAMIC REPUBLIC OF IRAN

## Abstract

**Introduction:**

HIV testing uptake remains very low among men who have sex with men (MSM) in Malaysia, a subgroup still bearing a disproportionate HIV burden. Therefore, we sought to identify and measure factors associated with never-testing for HIV among Malaysian MSM to further characterize this subgroup and inform future public health interventions addressing low testing rates in the country.

**Methods:**

We conducted a cross-sectional online survey among Malaysian MSM (August to September 2021). Participants completed questionnaires, including socio-demographic characteristics, HIV/STI testing practices, drug- and sex-related behaviors, and knowledge and attitudes toward HIV prevention services. Out of 1,799 completed surveys, 870 were included in the analysis after eliminating duplicates and incomplete surveys. We used logistic regression to determine factors associated with never-testing for HIV.

**Results:**

Overall, one-third of the study participants (33.8%) reported having never tested for HIV. Of those who reported to have tested for HIV (n = 576), half reported that they had tested for HIV in the past 6 months (50.3%). In multivariable logistic regression, MSM with HIV sero-concordant partner (aOR = 3.44, 95% CI = 1.56–7.60), without a prior diagnosis of a sexually transmitted infection (aOR = 2.83, 95% CI = 1.46–5.49), unaware of pre-exposure prophylaxis (PrEP; aOR = 2.71, 95% CI = 1.74–4.21), unaware of someone taking PrEP (aOR = 1.64, 95% CI = 1.15–2.35), and unwilling to use PrEP (aOR = 2.48, 95% CI = 1.43–4.30) had higher odds of never been tested for HIV. In contrast, MSM who were older (aOR = 0.95, 95% CI = 0.93–0.97) and of the Malaya ethnic group (aOR = 0.59, 95% CI = 0.37–0.95) had lower odds of never testing for HIV.

**Conclusion:**

Our findings shed light on the characteristics of HIV never-testers among MSM in Malaysia. The results indicate the need for innovative strategies to increase the uptake of HIV testing services among members of the MSM community.

## Introduction

Men who have sex with men (MSM) continue to face a disproportionate burden of HIV globally [1, 2], with MSM having 28 times the risk of acquiring HIV compared to the general population [3]. The issue is further exacerbated in low-or-middle-income countries, such as Malaysia, where MSM face severe implications for low testing rates, including late diagnosis, acute symptoms, limited access to care and treatment, and appropriate prevention. In Malaysia, the HIV epidemic is growing rapidly and is now the fifth largest in the Asia-Pacific region, with over 82,000 cases, with a notable shift toward impacting MSM. Between 2008 and 2018, MSM accounted for an increasing number of new HIV cases, from 10% to 21.6% [4]. This trend is predicted to continue, making MSM the key population with the highest HIV prevalence in Malaysia by 2030 [4]. Individuals who are unaware of their HIV status due to a lack of testing contribute significantly to new HIV infections. In addition to contributing to the increase of HIV incidence in Malaysia, especially in MSM who account for 63% of new HIV diagnoses in 2021, never testing for HIV also leads to delayed diagnosis of HIV with severe implications [4, 5]. A recent study revealed that individuals who were diagnosed with HIV at a late stage exhibited a higher odds of mortality compared to those who were diagnosed at earlier stage [6]. Late diagnosis of HIV has been shown to result in increased morbidity, mortality, and healthcare costs in previous studies [7].

HIV testing is a crucial step that serves as an entry point to the appropriate prevention, treatment and care services, and other clinical and support services [8]. The World Health Organization (WHO) recommends that individuals at increased risk for HIV acquisition get tested at least every 6 months [1, 2]. Malaysia has adopted guidelines for scaling up HIV testing, notably through a range of differentiated testing approaches, including facility-based testing, community-based testing, self-testing, and online platforms (e.g., website, app-based, social media) [8]. Despite these efforts, the HIV testing rates among Malaysian MSM continue to be suboptimal due to multi-level factors. Current research in Malaysia and ASEAN (Association of Southeast Asian Nations) countries posits that risk perception, fear and anxiety surrounding results, denial of symptoms, testing cost, anonymity, stigma and discrimination, and accessibility are barriers to HIV testing [9–13]. Moreover, Malaysia has outlawed same-sex sexual activities, contributing to the high stigmatization and discrimination against MSM. This dissuades them from pursuing HIV testing.

While most research focuses on risk factors associated with HIV infections, there is scant literature exploring the covariates of never testing for HIV, particularly in Malaysia. Therefore, we sought to investigate the risk factors associated with never testing for HIV among Malaysian MSM in hopes of better understanding and guiding HIV testing interventions in Malaysia.

## Methods

### Study design and sample

A cross-sectional, online survey was conducted from August to September 2021 assessing HIV testing practices among MSM in Malaysia. The eligibility criteria included: 1) being 18 years or older; 2) self-reporting HIV negative or HIV status unknown; 3) being a cis male who has sex with men; and 4) being able to read and understand English.

### Recruitment

Participants were recruited through convenience sampling via advertisements on social media (i.e., Facebook) and geosocial networking (GSN) app for gay men (i.e., Hornet). Messages in chat boxes were sent to all users in Malaysia on Hornet. At the same time, flyers were posted on the Facebook pages of non-governmental organizations and community-based organizations that provided services for Malaysian MSM. In addition, banner advertisements targeting MSM residing in Malaysia were used. Interested users who clicked on the advertisements were redirected to the eligibility screening tool, followed by a brief online consent form on Qualtrics. Eligible participants read and completed the consent form detailing the purpose, risks, and benefits of the study before completing the anonymous survey. On average, participants took 35 min to complete the online survey. The study protocol and the consent form were approved by the University of Malaya Research Ethics Committee (UMREC) and the Institutional Review Board at the University of Connecticut.

We followed established protocols for identifying and eliminating potential duplicate cases [14]. Specifically, possible duplicates were identified based on commonalities in age, sexual orientation, and ethnicity. All the cases with similar characteristics were examined manually by focusing on responses to other questions, such as education, income, relationship status, device and browser information, and completion duration.

A total of 1976 individuals accessed the survey. Of those, 1799 (91.0%) met the inclusion criteria and consented to participate in the survey. Of the 1799 who started the survey, 929 (51.6%) responses were excluded due to incomplete responses and missing data, thus leaving the final analytic sample to 870 (48.4%).

### Measures

The outcome variable, never testing for HIV, was measured using a single-item question, “*Have you ever been tested for HIV*?” The response was a dichotomous choice of “yes” or “no.” The variable was assessed against multiple independent variables, including demographic characteristics, psychological factors, sexual and drug risk behaviors, and PrEP knowledge and attitude.

### Demographic characteristics

Variables describing the demographic characteristics of the participants were collected, including age, ethnicity, sexual orientation, monthly income, educational status, and relationship status. Age was kept as a continuous variable. Monthly income was categorized to represent the poverty level, low-income, and middle-upper income ranges according to recent monthly income statistics in Malaysia [15]. Certain ethnic categories were combined as they were too small for comparison (N<5). The ethnicity categories were dummy-coded to clarify differences between groups in the bivariate analysis.

### Psychological factors

Participants were asked two questions about their experiences with depressive symptoms using the PHQ-2 scale, a two-item screening scale for depressive symptoms that ranges from 0–6 [16]. The score of 3 or higher on the PHQ-2 scale was categorized as depressive symptoms. Participants were also asked about experiencing stigma or discrimination from their healthcare providers. In addition to stigma in the healthcare field, we also asked participants about their stigma and acceptance experience by their family members. We asked participants about their status on “coming out” to their family members; those who reported disclosing their sexual orientation to a few or more family members were considered as “out”.

### Sexual and drug risk behaviors

Participants were asked about their sexual- and drug-related behaviors in the past 6 months. We asked participants if they engaged in anal sex with a man, had an HIV-positive sex partner, engaged in transactional sex (i.e., paid or been paid for sex), engaged in chemsex (i.e., use of psychoactive drugs before or during sexual activity), diagnosed with an STI (i.e., Chlamydia, Gonorrhea, Syphilis, Hepatitis B, and Hepatitis C), and injected drugs. All the variables were dichotomous and were kept as such in the analyses. In addition, a variable describing participant’s perception of contracting HIV in the next six months was added to this category. This variable was dichotomized, wherein those who reported extremely high likelihood were labeled as ‘*high’* and everyone else was coded as ‘*low*’.

### PrEP knowledge and attitude

Variables exploring the participant’s knowledge and overall mindset towards PrEP prior to the study were grouped in this category. Knowledge of PrEP was explored with two questions, while the attitude surrounding PrEP was measured using one question. All variables were dichotomous as well.

### Statistical analysis

Data analyses were performed using IBM SPSS v. 28 [17]. Descriptive statistics, including frequencies and percentages for categorical variables, were computed. Bivariate analysis followed by multivariable logistic regression analysis was performed to determine potential factors associated with the variable of interest (i.e., never testing for HIV), and an adjusted odd ratio at a 95% confidence interval was calculated. In the bivariate analyses, the multivariable regression model included variables found to be significant at a 10% level. The Variance Inflation Factor (VIF) was also computed to check the collinearity among predictor variables before these variables were included in the model.

## Results

Participant characteristics are described in Table 1. The participants’ mean (± SD) age was 32.1 (± 9.1) years. Two-thirds of the participants graduated from the university (65.9%), and half of the respondents belonged to the Chinese (49.2%) ethnic group. Most participants were single (70.7%) and had not disclosed their sexual orientation to their family (69.1%). Regarding sexual behaviors, over half of the participants reported having had anal sex in the past 6 months (62.3%), and of those (*n* = 542), more than half (330/542; 60.9%) reported engaging in condomless anal sex. A minority of participants reported having an HIV-positive sexual partner (9.9%), having been paid for sex (5.6%), and injected drugs (3.1%).

**Table 1:** Factors associated with lifetime HIV non-tester using bivariate logistic regression model.

Variable		N = 870	Lifetime HIV Non-Tester		OR (95% CI)	p-value
		n (%)	Yes n (%)	No n (%)		
			n = 294	n = 576		
**Demographic Characteristics**						
Age (M, SD)		870 (32.11, 9.09)			**0.935 (0.917, 0.953)**	**<0.001**
Ethnicity						
	Chinese	428 (49.2)	161 (54.8)	267 (46.3)	**1.401 (1.057, 1.857)**	**0.019**
	Indian	53 (6.1)	18 (6.1)	35 (6.1)	1.008 (0.561, 1.813)	0.979
	Malay	255 (29.3)	69 (23.5)	186 (32.3)	**0.643 (0.466, 0.887)**	**0.007**
	Other	134 (15.4)	46 (15.6)	88 (15.3)	1.029 (0.698, 1.516)	0.887
Sexual Orientation (Gay)						
	Yes	617 (70.9)	185 (62.9)	432 (75.0)	REF	REF
	No	253 (29.1)	109 (37.1)	144 (25.0)	**1.768 (1.306, 2.392)**	**<0.001**
Monthly Income						
	>2209 RM	603 (69.3)	166 (56.5)	437 (75.9)	REF	REF
	1170–2208 RM	121 (13.9)	51 (17.3)	70 (12.1)	**1.918 (1.282, 2.869)**	**0.002**
	<1169 RM	146 (16.8)	77 (26.2)	69 (12)	**2.938 (2.028, 4.256)**	**<0.001**
University Graduate						
	Yes	573 (65.9)	170 (57.7)	403 (70.0)	REF	REF
	No	297 (34.1)	124 (42.3)	173 (30.0)	**1.699 (1.269, 2.276)**	**<0.001**
In a relationship						
	Yes	255 (29.3)	68 (23.1)	187 (32.5)	REF	REF
	No	615 (70.7)	226 (76.9)	389 (67.5)	**1.598 (1.157, 2.205)**	**0.004**
**Psychological Factors**						
Disclosed sexual orientation to family						
	Yes	269 (30.9)	78 (26.5)	191 (33.2)	REF	REF
	No	601 (69.1)	216 (73.5)	385 (66.8)	**1.374 (1.006, 1.876)**	**0.046**
Depressive Symptoms						
	Yes	306 (35.2)	100 (34.0)	206 (35.8)	REF	REF
	No	564 (64.8)	194 (66.0)	370 (64.2)	1.080 (0.804, 1.451)	0.609
Ever experienced discrimination by healthcare provider						
	Yes	112 (12.9)	36 (12.2)	76 (13.2)	REF	REF
	No	758 (87.1)	258 (87.8)	500 (86.8)	1.089 (0.713, 1.665)	0.692
**Sexual and Drug Risk Behaviors**						
Engaged in anal sex with a man (Past 6 months)						
	Yes	542 (62.3)	153 (52.0)	389 (67.5)	REF	REF
	No	328 (37.7)	141 (48.0)	187 (32.5)	**1.917 (1.438, 2.556)**	**<0.001**
HIV-positive sexual partner (Past 6 months)						
	Yes	86 (9.9)	8 (2.7)	78 (13.5)	REF	REF
	No	784 (90.1)	286 (97.3)	498 (86.5)	**5.559 (2.666, 11.760)**	**<0.001**
Engaged in transactional sex (Past 6 months)						
	Yes	49 (5.6)	14 (4.8)	35 (6.1)	REF	REF
	No	821 (94.4)	280 (95.2)	541 (93.9)	1.294 (0.685, 2.445)	0.427
Engaged in chem sex (Past 6 months)						
	Yes	78 (9.0)	60 (10.4)	18 (6.1)	REF	REF
	No	792 (91.0)	516 (89.6)	276 (93.9)	**1.783 (1.032, 3.080)**	**0.038**
Condomless anal sex (Past 6 months)						
	Yes	658 (75.6)	236 (80.3)	422 (73.3)	REF	REF
	No	212 (24.4)	58 (19.7)	154 (26.7)	**0.673 (0.479, 0.947)**	**0.023**
Diagnosed with an STI (Past 6 months)						
	Yes	103 (11.8)	13 (4.4)	90 (15.6)	REF	REF
	No	767 (88.2)	281 (95.6)	486 (84.4)	**4.003 (2.197, 7.292)**	**<0.001**
Likelihood of contracting HIV in the next 6 months						
	High	41 (4.7)	7 (2.4)	34 (5.9)	REF	REF
	Low	829 (95.3)	287 (97.6)	542 (94.1)	**2.572 (1.126, 5.875)**	**0.025**
Injected drugs (Past 6 months)						
	Yes	27 (3.1)	7 (2.4)	20 (3.5)	REF	REF
	No	843 (96.9)	287 (97.6)	556 (96.5)	1.475 (0.616, 3.529)	0.383
**PrEP Knowledge and Attitude**						
Ever heard of PrEP						
	Yes	704 (80.9)	192 (65.3)	512 (88.9)	REF	REF
	No	166 (19.1)	102 (34.7)	64 (11.1)	**4.250 (2.983, 6.054)**	**<0.001**
Willingness to use PrEP						
	Yes	788 (90.6)	245 (83.3)	543 (94.3)	REF	REF
	No	82 (9.4)	49 (16.7)	33 (5.7)	**3.291 (2.064, 5.247)**	**<0.001**
Know of someone taking PrEP						
	Yes	421 (48.4)	96 (32.7)	325 (56.4)	REF	REF
	No	449 (51.6)	198 (67.3)	251 (43.6)	**2.671 (1.990, 3.584)**	**<0.001**

Numbers that are in boldface indicate significance of p<0.10.

SD denotes standard deviation.

M denotes the mean.

REF denotes reference group.

Over one-third (33.8%) of participants reported never having tested for HIV in their life. Of those who reported to have tested for HIV (n = 576), half reported that they had tested for HIV in the past 6 months (50.3%).

### Correlates of never testing for HIV

Table 1 shows the bivariate associations between demographic characteristics, psychosocial factors, PrEP knowledge and history, recent sexual and HIV-related behavior, and never testing for HIV. Table 2 shows independent correlates of never testing for HIV in the full multivariable logistic regression model. In multivariable logistic regression, MSM with HIV seroconcordant partner (aOR = 3.438, 95% CI = 1.555–7.601), without a prior diagnosis of a sexually transmitted infection (aOR = 2.834, 95% CI = 1.464–5.488), unaware of pre-exposure prophylaxis (PrEP; aOR = 2.705, 95% CI = 1.737–4.212), unaware of someone taking PrEP (aOR = 1.644, 95% CI = 1.150–2.349), and unwilling to use PrEP (aOR = 2.484, 95% CI = 1.434–4.301) had higher odds of never been tested for HIV. Whereas MSM who were older (aOR = 0.947, 95% CI = 0.927–0.967) and of the Malaya ethnic group (aOR = 0.589, 95% CI = 0.367–0.947) had lower odds of never testing for HIV.

**Table 2:** Factors associated with lifetime HIV non-tester using multivariate logistic regression model.

Variable		B	aOR (95% CI)	p-value
**Demographic Characteristics**				
Age		-0.055	**0.947 (0.927, 0.967)**	**<0.001**
Ethnicity				
	Malay	-0.529	**0.589 (0.367, 0.947)**	**0.029**
	Chinese	0.385	1.470 (0.966, 2.236)	0.072
Are you Gay?				
	Yes	REF	REF	REF
	No	0.314	1.369 (0.945, 1.984)	0.097
Income				
	>2209	REF	REF	REF
	1170–2208 RM	0.126	1.134 (0.703, 1.828)	0.606
	<1169 RM	0.199	1.220 (0.747, 1.992)	0.428
University Graduate				
	Yes	REF	REF	REF
	No	0.186	1.204 (0.837, 1.732)	0.317
In a relationship				
	Yes	REF	REF	REF
	No	0.317	1.373 (0.946, 1.993)	0.945
**Psychological Factors**				
Disclosed sexual orientation to family				
	Yes	REF	REF	REF
	No	0.264	1.302 (0.901, 1.882)	0.160
**Sexual and Drug Risk Behaviors**				
Engaged in anal sex with a man				
	Yes	REF	REF	REF
	No	-0.051	0.950 (0.648, 1.394)	0.794
HIV-positive sexual partner				
	Yes	REF	REF	REF
	No	1.235	**3.438 (1.555, 7.601)**	**0.002**
Engaged in chem sex				
	Yes	REF	REF	REF
	No	-0.114	0.892 (0.470, 1.694)	0.727
Condomless anal sex				
	Yes	REF	REF	REF
	No	-0.232	0.793 (0.513, 1.225)	0.296
Diagnosed with an STI				
	Yes	REF	REF	REF
	No	1.042	**2.834 (1.464, 5.488)**	**0.002**
Likelihood of contracting HIV in the next 6 months				
	High	REF	REF	REF
	Low	0.413	1.511 (0.565, 4.043)	0.411
**PrEP Outlook**				
Ever Heard of PrEP				
	Yes	REF	REF	REF
	No	0.995	**2.705 (1.737, 4.212)**	**<0.001**
Willingness to use PrEP				
	Yes	REF	REF	REF
	No	0.910	**2.484 (1.434, 4.301)**	**0.001**
Know of someone taking PrEP				
	Yes	REF	REF	REF
	No	0.497	**1.644 (1.150, 2.349)**	**0.006**

Numbers that are in boldface indicate significance of p<0.05.

REF denotes reference group.

## Discussion

The findings from this study provide empirical evidence on the factors associated with never testing for HIV among Malaysian MSM, a group who are at increased risk of HIV acquisition [1, 8]. Our findings corroborate previous research suggesting high rates of non-testing behavior despite engagement in high-risk behavior [9, 18–21]. As a result, Malaysia has yet to meet the UNAIDS goal of ending the HIV epidemic.

We found that men who did not have a known HIV-positive sexual partner had three times the odds of never testing than those who did. Since those participants knew they had never had sexual relations with an HIV-positive person, their perception of contracting HIV is low, hence the increased likelihood of never testing for HIV. This finding aligns with prior evidence that low perceived risk for HIV infection among Malaysian MSM was found to be associated with a lack of testing [22, 23]. Furthermore, other variables, including fear of positive results and denial, contribute to the lack of lifetime HIV testing [9, 12, 19, 22, 23]. This is highlighted further in our findings since participants who never tested for HIV had elevated odds of not being diagnosed with an STI, meaning they possibly could have STI infections given their high-risk behavior but have yet to test for STIs possibly out of the fear of positive results and/or symptom denial. Efforts to decrease stigma around HIV and STIs could decrease the stigma around STI testing in general.

Mandatory HIV testing in the Malaysian Muslim community was implemented in 2009 [13, 24]. As a result, individuals from ethnically Muslim households, which tend to be Malay, are mandated to test for lifetime HIV [18, 24, 25]. This mandatory testing basis is directly related to our finding of decreased likelihood of being an HIV non-tester among participants who identified as having Malay for their ethnic background. The issue of mandatory testing among the Malay Muslim community is also controversial with organizations like the WHO and UNAIDS, as they have strong reservations since it does not follow the 5C’s of testing for HIV prevention [24]. The ethnic difference in our findings is worrisome as it shows that MSM of differing cultural backgrounds who do not perceive lifetime HIV testing as mandatory are more likely to have never tested despite their risk. While compulsory testing does affect the testing data, efforts to encourage HIV testing should not be forced. Instead, increasing education and awareness around the benefits of HIV testing should be promoted. In addition, decreasing the stigma around HIV and STI testing aids in the WHO’s long-term goal while encouraging both Muslims and non-Muslims to participate in voluntary testing.

Younger MSM have historically been shown to have higher rates of never testing for HIV [12, 19, 20, 26]. We found that never testing for HIV significantly decreased with age, meaning younger participants had higher odds of never testing. In addition to low-risk perception, fear of positive results, and lack of privacy and accessibility, younger MSM have also reported having little knowledge of HIV prevention efforts and the severe consequences of late diagnosis [9, 12, 19, 23, 26, 27]. Our findings add further evidence that youth MSM experience elevated odds of never testing for HIV despite the need to. Furthermore, previous findings show that delayed testing is a substantial issue with early diagnosis of HIV [27]. While internalized factors such as self-perception or stigma are associated with the delay in testing, the lack of education and social stigma also play a role. Literature exploring AIDS/HIV-related stigma suggests that more efforts have been put into interventions aimed at addressing internalized stigma than those that address the social stigma and education surrounding HIV testing in Malaysia [27, 28]. Our findings reiterate previous postulates stating the lack of programs and interventions that address social and structural differences like ethnicity and age in Malaysian MSM are associated with never testing for HIV [9, 19, 21, 22].

We found that participants who were unaware of PrEP or were not willing to take PrEP had higher odds of never testing for HIV. This may go back to the perceived low risk of HIV infection and, therefore, being unaware of HIV services. HIV testing serves as an entry point to HIV prevention and treatment services. However, ignorance around the role of testing leads to a lower likelihood of non-testing. In addition, issues surrounding HIV stigma and discrimination are plausible for never testing for HIV and, therefore, being unaware of HIV preventative measures like PrEP among MSM. Strategies targeting HIV prevention efforts, including PrEP education and campaigns to reduce the stigma surrounding it, are crucial for decreasing the non-tester rates to meet the WHO’s 95-95-95 objective.

Our study suggested a number of factors associated with never testing for HIV among Malaysian MSM, which indicate the need for tailored campaigns to make MSM aware of HIV services are necessary. Reaching out via social media platforms, online services, and digital platforms to target younger MSM while also putting efforts into community-based and key population-led operations are necessary steps to help address the gaps in HIV testing. Furthermore, there is a need for a range of testing approaches to reach MSM living with HIV who do not know their status and others at risk of acquiring HIV. Self-testing is a new and private method to test for HIV that can be used to avoid the social fears surrounding testing [10]. Likewise, incorporating newer concepts like ’Undetectable = Untransmittable’ (U = U) and preventive treatments such as a single dose of 200 mg doxycycline within 72 hours into awareness campaigns can encourage MSM individuals to test for both HIV and STIs. Not one single modality is going to be an all-encompassing successful solution for HIV non-testing among the entirety of the Malaysian MSM population. Therefore, adapting a mix of various approaches and models, like the Differentiated Service Delivery Models (DSD), is a more effective and better solution to address HIV testing gaps [29–31]. Differentiated testing models that include the best mix of testing services—such as self-testing, index testing and various community and facility-based testing services—are suitable for various settings. In the case of self-testing, Malaysian MSM reported a high acceptability for it when presented with a scenario where the testing service had no cost, high accuracy, and anonymity [10]. Given our findings, having a low-cost, accurate, and private way to test for HIV would encourage Malaysian MSM, especially the young, to know their status. The employment of DSD, a person-centered approach to HIV services, including testing, prevention, and treatment among Malaysian MSM, provides promising success in improving the rates of HIV non-testing [29–31].

### Strengths

Our study had many notable strengths, firstly addressing the multiple factors related to HIV testing among Malaysian MSM. Our findings add to previous literature regarding barriers to HIV testing both globally and in Malaysia. Unlike most literature, our study presents quantitative data showing the correlation of never testing for HIV among Malaysian MSM. In addition, the data highlights troubling findings like delays in testing and ethnic disparities that are associated with lifetime HIV testing. Finally, our study highlights the need for more programs that target social and structural barriers to HIV testing in Malaysia, as most target personal stigma.

### Limitations

Our findings should be interpreted with certain limitations to consider. First, due to the cross-sectional nature of this study, we cannot establish a causal relationship between lifetime HIV never testing and other significantly associated variables. Second, our study focused exclusively on Malaysian MSM, limiting the generalizability of our findings to other populations or contexts. Third, the study utilized an online survey, leading to possible gaps in the questions’ interpretability and potential desirability bias from the participants.

## Conclusion

Our findings add to the current body of literature that shed light on the characteristics of HIV never-testers among MSM in Malaysia. The findings indicate there is a need for innovative strategies, such as using self-testing, online testing applications, peer-based models, expanding outreach and education efforts and community-led models to scale up HIV testing among MSM in Malaysia and elsewhere.
